# Vitamin D in Health and Disease

**DOI:** 10.3390/biomedicines11010010

**Published:** 2022-12-21

**Authors:** Giuseppe Murdaca, Sebastiano Gangemi

**Affiliations:** 1 Department of Internal Medicine, University of Genova, 16132 Genova, Italy; 2 Department of Internal Medicine, IRCCS Ospedale Policlinico San Martino, 16132 Genova, Italy; 3 Department of Clinical and Experimental Medicine, School and Operative Unit of Allergy and Clinical Immunology, University of Messina, 98125 Messina, Italy

Vitamin D (VD) is a fat-soluble hormone that plays a fundamental role not only in calcium homeostasis and bone metabolism, but also has anti-inflammatory and antioxidant properties, acting on both innate and adaptive immunity. Therefore, it is now scientifically considered an immunomodulating agent. It has been shown that VD and microbiomes act in concert and direct the etiopathogenesis and evolution of many allergic diseases. Among the many activities that are in current times attributed to VD, it is necessary to remember the antiproliferative, antiangiogenic and prodifferentiation activity that, undoubtedly, attributes to VD itself, playing a role in cancer carcinogenesis that has not yet been completely gutted. However, it would appear that some of its carcinogenic effects may be attributed to binding to its receptor (VDR) and the subsequent modulation of tumor miRNA expression. In this Editorial, I would like to briefly summarize the main concepts described in the eight papers published in the Special Issue “Vitamin D in Health and Disease”. The first impression that came from reading the titles of the articles is that VD is implicated in the pathogenesis of both chronic respiratory and cardiovascular diseases. It goes without saying that it is likely that VD acts in the development and clinical evolution of many chronic immune-mediated diseases, including allergic ones. The authors of the various papers underlined the association between low serum levels of VD that favor release, and Th2 cells of proinflammatory cytokines, including IL-4, IL-5 and IL-13, which might guide the inflammatory state at the base of many chronic diseases. The role of these cytokines in chronic rhinosinusitis (CRS), which is characterized by a diffuse inflammation of the mucosa, whose etiopathogenesis is not yet fully understood, has now been widely demonstrated [1]. It should be remembered that CRS is classified into two subtypes, depending on the presence or absence of nasal polyposis (CRSwNP and CRSsNP, respectively). Notably, IL-4, IL-5 and IL-13 seem to direct the development of CRSwNP, while, on the contrary, IFN-γ released by Th1 cells, would favor the development of CRSsNP [2,3]. Several studies confirmed that low VD levels could promote an increased cytokine release from inflammatory cells and fibroblasts [3], perpetuating chronic inflammatory sinus diseases and the degree of severity of NP. In particular, I would like to underline the work of Christensen et al. [4], who confirmed how maintaining VD serum levels allows to control the secretion of IL-4, IL-5 and IL-13, and, at the same time, induce the production of IL-10 at the level of sinuses, with the consequent inhibition of inflammation. Furthermore, Camargo et al. [5] confirmed the importance of VD supplementation to prevent the winter exacerbation of atopic dermatitis. Confirming the importance of bringing VD levels back to adequate values, Afzal et al. [6] discuss the therapeutic potential of VD in inflammatory lung diseases (ILDs), including asthma, lung cancer, bronchiectasis, pulmonary fibrosis, acute respiratory distress syndrome and *chronic obstructive pulmonary disease* (COPD), in which inflammation represents a significant component of the disease [7]. On the other hand, it is well established that the insufficiency of calcitriol supports the inflammatory process in COPD. Therefore, maintaining physiological levels of VD allows to exploit the anti-inflammatory action of VD itself. Notably, VD carries out its anti-inflammatory activity by acting on cellular elements that play a role in inflammatory processes. Macrophages, an important cell type in the innate immune system, express the VD receptor (VDR). There are bacterial strains capable of regulating the expression of VDR and, consequently, the signals that derive from the VD/VDR interaction. In particular, *M. tuberculosis* activates the expression and activity of VDR and CYP27B1 by inducing the intracellular signal capable of stimulating the synthesis of cathelicidin, which favors the death of *M. tuberculosis*. It should be remembered that VD inhibits endoplasmic reticulum stress by counterbalancing the stimulating activity on macrophages by IFN-γ, as well as inhibiting the proliferation and activity of human dendritic cells (DCs). In summary, RV can modulate innate immune responses [6]. There are now scientific data confirming the importance of VD in improving the clinical picture in asthmatic patients. On the other hand, the anti-inflammatory action of RV is also expressed through its ability to reduce the serum levels of IL-17A and increase the levels of IL-10, as demonstrated in patients with asthma. RV has been shown to inhibit the profibrotic action of TGF-b1 in patients with pulmonary fibrosis [6,8]. Oriano et al. [9] confirmed that VD-binding protein (DBP) polymorphisms and the resulting isoform are involved in the pathogenesis and severity of bronchiectasis, where DBP is coded by the GC gene. Notably, the GC1f isoform (rs7041/rs4588 A/G) correlated with a more severe disease, a higher incidence of chronic infections and a lower bronchiectasis etiology and comorbidity index (BACI) score. On the other hand, the GC1s isoform had a milder phenotype with increased VD levels and a lower comorbidities score.

Endothelial cells line the inner lumen of all vessels while preserving the integrity of the vascular system. Endothelial dysfunction participates in the phenotypic expression of accelerated atherosclerosis and, therefore, in the increased risk of cardiovascular diseases. VD deficiency is associated with endothelial dysfunction, partially because of the downregulation of the VDR [10]. A significant number of studies have correlated vitamin D deficiency with an increased risk of developing heart arrhythmias, arterial hypertension, diabetes mellitus and sudden cardiac death [11,12]. Scrimieri et al. [13] investigated the potential protective role of VD on human umbilical vein endothelial cells (HUVECs). The increase in lipogenesis with a greater quantity of available triglycerides and the reduced oxidation of fatty acids favor the negative evolution of endothelial dysfunction. Furthermore, the glucose-induced thioredoxin-interacting protein (TXNIP) upregulation induces the accumulation of reactive oxygen species and, as a consequence, of lipid droplets. Low VD levels favor the development of coronary artery calcifications, the onset of atrial fibrillation (AF) and heart failure, while the downregulation of CYP27B1 (1-hydroxylase) has been associated with the severity of cardiovascular diseases [14]. Notably, if the serum levels of RV are not sufficient, they can affect cardiac electrical activity with consequent negative impacts on ventricular repolarization with the risk of the onset of severe arrhythmias [15]. Therefore, Barsan et al. [16] focused on the need to periodically monitor VD levels to choose the appropriate time for VD supplementation and the achievement of protective physiological levels. In support of this goal, Scrimieri et al. [13] demonstrated that adequate serum levels of VD downregulated TXNIP by preventing oxidative stress and by correcting the lipid metabolism and storage in lipid droplets, thus, restoring endothelial function. Marino et al. [17] demonstrated that the antiatherogenic protective effect of VD could be ascribable to the regulation of proteins involved in lipid transport and clearance. Indeed, VD decreases fatty acid accumulation in THP-1-derived macrophages exposed to an excess of free fatty acid (FFA). In particular, the addition of VD increases peroxisome proliferator-activated receptor gamma (PPAR)-γ1 levels, CPT-1A and ABCA1 proteins, while, by contrast, it blocks the increase in both CD-36 and C/EB proteins induced through FFA administration. Abulmeaty et al. [18] reported that VD deficiency is frequently present in heart transplant (HT) patients, but a 2-year supplementation of VD did not seem to have a positive impact on the bone mineral density (BMD). Furthermore, obesity represents an independent risk factor for the onset of cardiovascular diseases, for which vitamin D levels have not appeared to have any positive impact. Confirming this, VD supplementation failed to reduce the incidence rate of cardiovascular diseases and mortality. The link between VD and the composition of the gut microbiome is well established. Singh et al. [19] demonstrated that low VD levels induced changes in the composition of the gut microbiome in children, dominated by *Prevotella* as opposed to *Bacteroides*. The authors suggest to consider that host genetics and baseline gut microbiota compensate in interpreting the VD status and designing better personalized strategies for therapeutic interventions. Eight papers developing the role of VD in cardiovascular and airway diseases were published in this Special Issue. The general conclusion would confirm that low levels of VD and alterations in the VD/VDR signal and in the microbiome are, undoubtedly, able to influence the immune system, supporting the pathogenesis of cardiovascular diseases and chronic inflammatory processes affecting the airways, including NP, COVID-19, asthma, lung cancer, COPD, bronchiectasis, pulmonary and cystic fibrosis, pneumonia and tuberculosis. It should be emphasized that the microbiome and RV deeply influence each other. In particular, RV levels may represent a risk factor for the increased incidence of respiratory tract infections, as demonstrated in childhood and adolescence. Finally, VD represents a valid aid to control the redox balance and reduce the onset of endothelial dysfunction and, thus, of accelerated atherosclerosis. Therefore, the maintenance of optimal VD serum levels could represent a relevant strategy for reducing the risk of cardiovascular and airway diseases.
